# Macrophage Activation Syndrome in Pediatric Systemic Lupus Erythematosus: A Systematic Review of the Diagnostic Aspects

**DOI:** 10.3389/fmed.2021.681875

**Published:** 2021-06-04

**Authors:** Altynay Abdirakhmanova, Vitaliy Sazonov, Zaure Mukusheva, Maykesh Assylbekova, Diyora Abdukhakimova, Dimitri Poddighe

**Affiliations:** ^1^Department of Medicine, Nazarbayev University School of Medicine (NUSOM), Nur-Sultan, Kazakhstan; ^2^Department of Biomedical Sciences, Nazarbayev University School of Medicine (NUSOM), Nur-Sultan, Kazakhstan; ^3^Program of Pediatric Rheumatology, Department of Pediatrics, National Research Center for Maternal and Child Health, University Medical Center, Nur-Sultan, Kazakhstan; ^4^Department of Pediatrics, National Research Center for Maternal and Child Health, University Medical Center, Nur-Sultan, Kazakhstan

**Keywords:** Macrophage Activation Syndrome, pediatric Systemic Lupus Erythematosus, diagnostic criteria, ferritin, hyper-ferritinemia

## Abstract

Macrophage Activation Syndrome (MAS) is a very severe complication of different rheumatic diseases, including pediatric Systemic Lupus Erythematosus (pSLE). MAS is not considered as a frequent complication of pSLE; however, its occurrence could be under-estimated and the diagnosis can be challenging. In order to address this issue, we performed a systematic review of the available medical literature, aiming to retrieve all those papers providing diagnostic (clinical/laboratory) data on patients with pSLE-related MAS, in individual or aggregated form. The selected case reports and series provided a pool of 46 patients, accounting for 48 episodes of MAS in total. We re-analyzed these patients in light of the diagnostic criteria for MAS validated in systemic Juvenile Idiopathic Arthritis (sJIA) patients and the preliminary diagnostic criteria for MAS in pSLE, respectively. Five clinical studies were also selected and used to support this analysis. This systematic review confirms that MAS diagnosis in pSLE patients is characterized by several diagnostic challenges, which could lead to delayed diagnosis and/or under-estimation of this complication. Specific criteria should be considered to diagnose MAS in different rheumatic diseases; as regards pSLE, the aforementioned preliminary criteria for MAS in pSLE seem to perform better than the sJIA-related MAS criteria, because of a lower ferritin cut-off.

## Introduction

Systemic Lupus Erythematosus (SLE) is a challenging autoimmune disease, whose clinical expression is widely variable: indeed, all organs and systems may be potentially affected (1). Worldwide, SLE incidence varies from 1 to 10 per 100,000 person-year, and the prevalence is reported to range from 20 to 70 per 100,000 persons. Both incidence and prevalence resulted to be higher in non-Caucasian population. Importantly, SLE is characterized by a clear gender predilection (female/male ratio up to 9:1), which is more pronounced than in other autoimmune diseases (2, 3).

Pediatric SLE (pSLE) is diagnosed in patients younger than 18 years: it represents 10–20% of all SLE cases and shows a variable prevalence of 1.89–25.7 per 100,000 children, according to the ethnic group. pSLE is characterized by female predominance (gender ratio of 4–5:1) and may have a more aggressive clinical course than adult SLE, especially in terms of neurological and renal manifestations (4–7).

In addition to several and heterogeneous organ-related and long-term complications, SLE patients may also develop the so-called Macrophage Activation Syndrome (MAS) (8, 9). MAS is a very severe, acute and potentially life-threatening condition, which is classified as a secondary form of hemophagocytic lymphohistiocytosis (HLH). Primary HLH is substantially caused by genetic defects leading to the impairment of the T and/or NK cells cytotoxic activity. This immunological aspect is also the main pathogenic event of secondary or acquired HLH, namely MAS, which may be due to a variable combination of factors, including the iatrogenic immunosuppression and/or intrinsic immune dysfunction related to the underlying rheumatic disease itself. Whenever one or more infectious agents (often viruses) cannot be efficiently cleared in rheumatic patients, the persistent activation of the CD8^+^ T cell/macrophage immune loop can result in uncontrolled systemic inflammation and, thus, massive production of several inflammatory cytokines (“cytokine storm”), leading to dysregulated hemophagocytosis in multiple organs (10).

The exact prevalence of MAS in pSLE patients is difficult to say, but may be under-estimated; however, a timely and certain diagnosis of MAS is a medical priority, especially in children affected with pSLE: indeed, the mortality rate is calculated to be around 5%, which is much higher than that in SLE patients without MAS (0.2%) (11). However, MAS diagnosis can be challenging, especially if it occurs in patients without any clear diagnosis of rheumatic disease. In general, the clinical manifestations can include persistent high-grade (sepsis-like) fever, hepatosplenomegaly, lymphadenopathy, and central nervous dysfunction (8, 10).

The European League Against Rheumatism and the American College of Rheumatology (EULAR/ACR) developed a set of criteria for the diagnosis of MAS in patients affected with JIA (12). However, there are no definitive MAS diagnostic criteria specific for other pediatric rheumatic diseases, including pSLE; therefore, the EULAR/ACR criteria are variably used by the clinicians to pursue a diagnosis of MAS in different clinical settings, but it is still unclear whether they can be safely and appropriately applied to diseases other than JIA.

Through this systematic review, we aim at analyzing and discussing the most relevant diagnostic aspects of MAS in pSLE patients.

## Materials and Methods

### Protocol

The PRISMA guidelines were used for this systematic review. This systematic review includes original articles and case reports/series, providing (diagnostic) information on pSLE patients developing MAS. Indeed, the primary aim of this systematic review is to assess the potential performance of the most used diagnostic criteria for MAS in this specific rheumatological setting, namely pSLE.

### Search Strategy

In order to retrieve all the original articles focused on pSLE complicated by MAS, a systematic literature search was conducted through PubMed, Scopus, and Web of Science databases by using the following keywords/terms: (systemic lupus erythematosus AND/OR SLE AND/OR pediatric SLE AND/OR juvenile SLE AND/OR childhood-onset SLE AND/OR children) AND (macrophage activation syndrome AND/OR MAS). The study period was from 2000 until March 15th, 2021.

**Figure 1:**
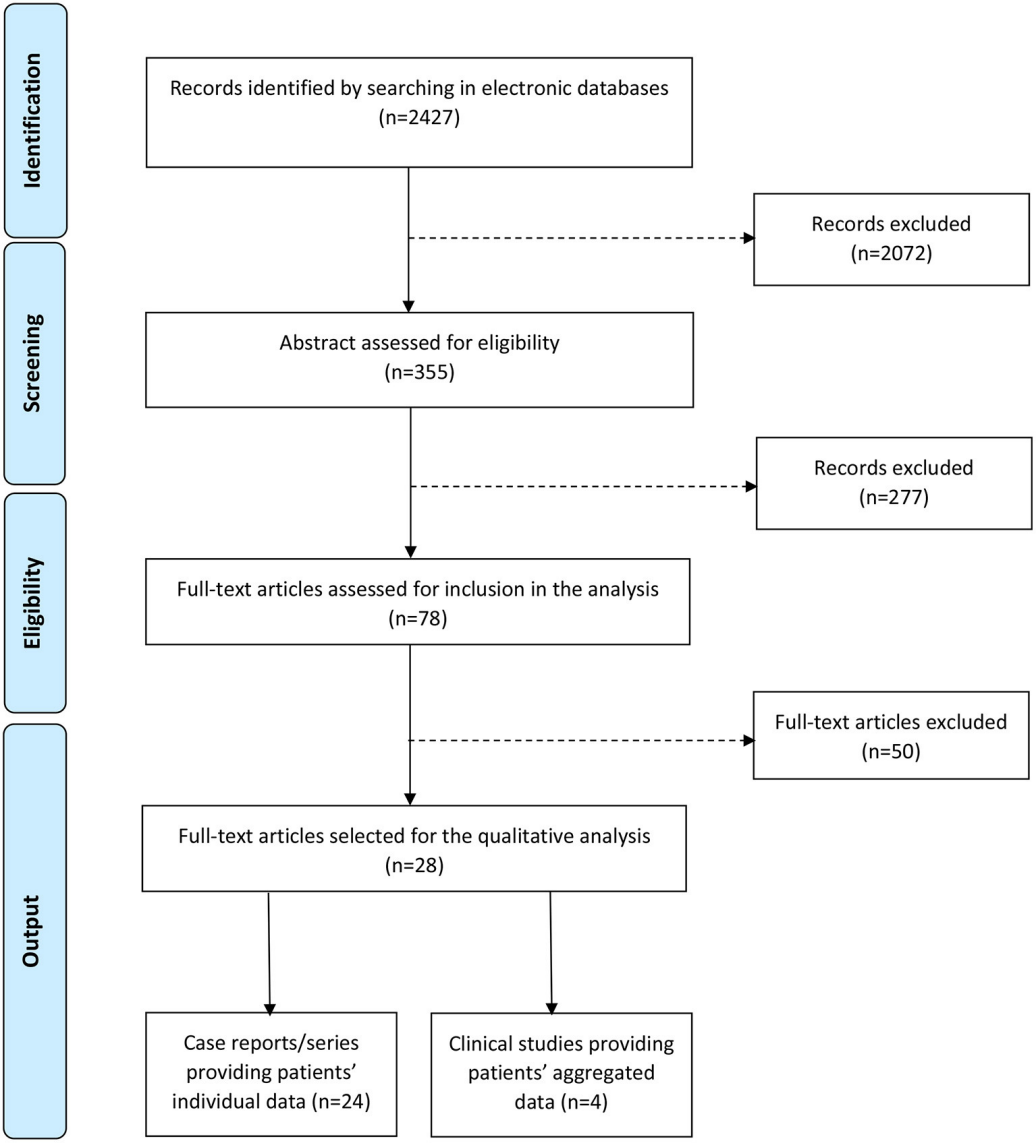
PRISMA flow diagram of the systematic literature research.

After screening a total of 2,427 items retrieved from the medical literature in the aforementioned electronic databases, duplicated records, review articles, abstracts and conference papers were eliminated; moreover, only publications in English language were considered. Therefore, after eliminating 2,072 items through this initial screening, 355 titles were considered for eligibility, based upon the abstract: 78 full-text papers were retrieved in order to identify all the original publications (case-control, cross-sectional, retrospective studies, case reports, and case series), describing only patients with pSLE-related MAS and providing diagnostic (clinical/laboratory) data in individual or aggregated form. This systematic literature search was carried out according to PRISMA guidelines, as schematically represented in Figure 1.

### Data Extraction

After a critical reading of the articles, data extraction was done by one investigator and was checked by a second investigator following these main inclusion criteria: any original articles including patients with pSLE-related MAS and providing diagnostic (clinical/laboratory) data in individual or aggregated form. In detail, the following items were extracted from each case report/series: first author's last name, publication year, patient's age and gender, temporal relationship between pSLE and MAS diagnoses, personal history of fever of unknown origin (FUO), qualitative therapy, outcome, laboratory parameters (hemoglobin, white blood cells, neutrophils, thrombocytes, presence of hemophagocytosis at histopathological analysis of the bone marrow, plasmatic sodium, serum ferritin, triglycerides, erythrocyte sedimentation rate, C-reactive protein, lactate dehydrogenase, aspartate transaminase, alanine aminotransferase, fibrinogen).

## Results

The final output of this systematic literature search consisted of 28 papers, including case reports/series (*n* = 24) and clinical studies (*n* = 4). The former type of articles provided individual clinical and laboratory data of patients affected with the pSLE-related MAS (13–36), whereas the clinical studies provided these data in aggregated form (11, 37–39). One article provided both types of data: individual information for pSLE-related MAS patients and aggregated values (which were compared to a control group of pSLE patients without MAS) (33).

In detail, all the selected case reports and series provided a pool of 46 patients with pSLE and a total of 48 episodes of MAS (indeed, two patients developed MAS twice). The main clinical aspects of these children (age, gender, temporal relationship between MAS occurrence and pSLE diagnosis, therapy and outcome) are presented in Table 1. The most relevant laboratory parameters (hemoglobin, platelets, white blood cells, neutrophils, ferritin, triglycerides, ESR, CRP, LDH, AST, ALT, fibrinogen, plasmatic sodium) for the diagnostic definition of MAS are summarized in Table 2.

**Table 1:** Demographics, treatment, and outcome of patients with pediatric Systemic Lupus Erythematosus (pSLE) and Macrophage Activation Syndrome (MAS) in case reports/series.

**References**	**Patient**	**Age (years)**	**Gender**	**SLE diagnosis at MAS onset**	**SLE duration before MAS**	**FUO**	**Therapy**	**Outcome**
Avčin et al. (13)	1	15	F	N	–	Y	MP	Fatal
McCann et al. (14)	2	10	F	N	–	Y	MP, PSL, CYC	Recovery
Rajam et al. (15)	3	14	F	N	–	Y	MP	Recovery
Yeap et al. (16)	4	14	F	N	–	Y	CSA, PSL	Recovery
Zulian et al. (17)	5	10	F	N	–	Y	MP, PRED	Recovery
Campos et al. (18)	6	17.9	F	Y	n/a	Y	MP	Fatal
	7	13.9	F	N	–	Y	MP, CYC	Recovery
	8	8.8	F	N	–	Y	MP, CYC	Fatal
	9	10.2	F	N	–	Y	MP	Recovery
	9*	13.2	/	Y	37 mo	Y	MP, CSA	Recovery
	10	10.8	F	N	–	Y	MP	Recovery
	11	14.6	F	Y	57 mo	Y	PRED, MMF	Fatal
	12	14.8	M	N	–	Y	MP	Recovery
	13	11.2	F	N	–	Y	MP	Recovery
	14	11.5	F	Y	3 mo	Y	MP, CYC	Recovery
	15	13.2	F	Y	41 mo	Y	MP, CSA, MMF	Fatal
Gharib et al. (19)	16	6	M	N	–	Y	MP, CSA	Recovery
Lin et al. (20)	17	16	F	Y	38 mo	Y	PSL	Recovery
	18	12	F	Y	32 mo	Y	MP	Fatal
Vilaiyuk et al. (21)	19	14	M	N	–	Y	MP	Recovery
	19*	14.5	/	Y	0.5 mo	Y	MP, CSA	Recovery
Jiménez et al. (22)	20	7	F	N	–	Y	MP, CSA, CYC	Recovery
Nakagashi et al. (23)	21	15	F	N	–	Y	MP, CSA, DexP	Recovery
Noh et al. (24)	22	14	F	N	–	Y	PSL, Deflazacort	Recovery
Gupta et al. (25)	23	10	F	Y	36 mo	Y	MP, PRED, CSA	Recovery
	24	15	F	N	–	Y	MP, CSA	Recovery
Alkoht et al. (26)	25	9	F	N	–	Y	MP, PSL, CYC	Recovery
	26	4	F	N	–	Y	MP, PSL, CYC	Recovery
Casciato et al. (27)	27	15	F	N	–	Y	MP, CSA	Recovery
Guru et al. (28)	28	15	F	N	–	Y	MP, CSA, HCQ	Recovery
Moideen et al. (29)	29	17	F	N	–	Y	MP	Recovery
Sowithayakasul et al. (30)	30	13	F	N	–	Y	MP, ETO, DEXA	Recovery
Aguirre-Martinez et al. (31)	31	9	M	N	–	Y	DEXA, CSA	Recovery
Gliwinska et al. (32)	32	9	F	N	–	Y	MP, CYC	Recovery
Sato et al. (33)	33	12	F	n/a^#^	–	Y	GC, MMF	Recovery
	34	7	F	n/a^#^	–	Y	GC, CYC, MP	Recovery
	35	12	M	n/a^#^	–	Y	GC, MMF, MP	Recovery
	36	13	F	n/a^#^	–	Y	GC, MMF, MP	Recovery
	37	13	M	n/a^#^	–	Y	GC, MP, CYC	Recovery
	38	14	F	n/a^#^	–	Y	GC	Recovery
	39	12	F	n/a^#^	–	Y	GC, AZA	Recovery
	40	14	F	n/a^#^	–	Y	GC	Recovery
	41	17	F	n/a^#^	–	Y	GC, MMF, MP	Recovery
	42	7	F	n/a^#^	–	Y	GC, MP	Recovery
	43	13	F	n/a^#^	–	Y	GC, MP	Recovery
Lin et al. (34)	44	11	F	N	–	Y	MP, PSL, CYC	Recovery
Surendran et al. (35)	45	12	M	N	–	Y	MP, PSL	Recovery
Cintron et al. (36)	46	16	M	N	–	Y	MP, HCQ	Recovery

*FUO, fever of unknown origin; SLE, Systemic Lupus Erythematosus; MAS, Macrophage Activation Syndrome; Y, yes; N, no; n/a, not available; –, not applicable; M, male; F, female; m, months; DEXA, dexamethasone; MP, methylprednisolone; PSL, prednisolone; CYC, cyclophosphamide; CSA, cyclosporine A; PRED, prednisone; MMF, mycophenolate mofetil; DexP, dexamethasone palmitate; HCQ, hydroxychloroquine; GC, glucocorticoid; AZA, azathioprine; ETO, etoposide*.

9^*^ second episode of MAS for the ninth patient;

19^*^ second episode of MAS for the nineteenth patient.

# *The authors state that during the study period 46 “new-onset” pSLE patients were admitted to their hospital and these 11 MAS patients belonged to this cohort*.

**Table 2:** Laboratory values of patients with pediatric Systemic Lupus Erythematosus (pSLE) and Macrophage Activation Syndrome (MAS) described in case reports/series.

**References**	**Pt**.	**Hb (g/L)**	**WBC (10^**9**^/L)**	**NEU (10^**9**^/L)**	**PLT (10^**9**^/L)**	**BM (HPC)**	**Sodium (mmol/L)**	**Ferritin (ng/mL)**	**Triglycerides (mg/dL)**	**ESR (mm/h)**	**CRP (mg/L)**	**LDH (U/L)**	**ALT (U/L)**	**AST (U/L)**	**Fibrinogen (mg/dL)**
Avčin et al. (13)	1	115	3.1	n/a	232	NEG	n/a	>1,000	n/a	28	n/a	2,740	99	196	n/a
McCann et al. (14)	2	75	1.6	0.8	83	NEG	n/a	2,087	n/a	31	<7	1,289	44	n/a	n/a
Rajam et al. (15)	3	90	4.8	n/a	n/a	POS	n/a	n/a	n/a	78	35	n/a	N/A	n/a	n/a
Yeap et al. (16)	4	84	2.4	1.08	46	NEG	n/a	73,968	297.3	n/a	n/a	2,675	375	2,534	14,100
Zulian et al. (17)	5	78	1.03	n/a	40	NEG	n/a	2,508	225.6	57	140	n/a	55	76	80
Campos et al. (18)	6	86	0.04	n/a	8	POS	n/a	1,890	349.5	n/a	n/a	n/a	98	46	n/a
	7	87	3.4	n/a	105	NEG	n/a	n/a	304.4	n/a	n/a	n/a	348	1,315	n/a
	8	114	3.7	n/a	64	NEG	n/a	n/a	781.4	n/a	n/a	n/a	458	1,667	n/a
	9	86	4.2	n/a	80	NEG	n/a	n/a	802.6	n/a	n/a	n/a	371	1,542	n/a
	9^*^	76	1.2	n/a	95	POS	n/a	27,602	330.9	n/a	n/a	n/a	99	229	n/a
	10	98	5.4	n/a	38	NEG	n/a	n/a	394.7	n/a	n/a	n/a	155	23	n/a
	11	96	7.1	n/a	300	NEG	n/a	1,718	525.6	n/a	n/a	n/a	215	939	n/a
	12	87	2.8	n/a	176	NEG	n/a	n/a	312.4	n/a	n/a	n/a	237	1,119	n/a
	13	85	2.2	n/a	111	NEG	n/a	n/a	81.42	n/a	n/a	n/a	44	82	n/a
	14	72	2.8	n/a	95	POS	n/a	3,079	624.8	n/a	n/a	n/a	152	375	n/a
	15	72	0.12	n/a	64	NEG	n/a	2,896	297.3	n/a	n/a	n/a	22	25	n/a
Gharib et al. (19)	16	59	0.48	0.05	293	NEG	127	8,654	397.3	148	48	705	24	57	1,300
Lin et al. (20)	17	49	0.6	0.42	74	POS	n/a	n/a	358.4	25	9	2,393	n/a	25	n/a
	18	62	0.7	n/a	12	POS	n/a	n/a	177.8	n/a	<4	727	n/a	127	n/a
Vilaiyuk et al. (21)	19	88	2.1	1.248	88	POS	n/a	>30,000	600	10	n/a	1,065	233	1,050	128
	19^*^	98	0.73	n/a	364	POS	n/a	>40,000	504.4	16	12	855	676	480	n/a
Jiménez et al. (22)	20	63	1.98	0.91	44	n/a	n/a	n/a	n/a	n/a	50.4	n/a	n/a	n/a	368
Nakagashi et al. (23)	21	n/a	n/a	n/a	79	POS	n/a	20,417	n/a	n/a	n/a	1,259	n/a	153	n/a
Noh et al. (24)	22	85	0.56	n/a	51	NEG	n/a	>1,650	281.4	n/a	n/a	794	n/a	451	n/a
Gupta et al. (25)	23	80	1	0.33	80	POS	n/a	45,395	349.5	70	15	740	550	640	n/a
	24	90	2.6	n/a	100	NEG	n/a	8,440	417.7	113	n/a	1,683	n/a	544	n/a
Alkoht et al. (26)	25	78	3.6	n/a	78	POS	n/a	591	281.4	n/a	n/a	606	n/a	NR	559
	26	82	1.9	n/a	173	POS	n/a	12,000	n/a	85	20	1,279	n/a	163	546
Casciato et al. (27)	27	100	1.9	0.95	4.1	POS	n/a	22,295	376.1	103	3.2	1,509	56	216	n/a
Guru et al. (28)	28	91	2.1	n/a	45	POS	n/a	>15,000	592	35	n/a	n/a	205	202	28
Moideen et al. (29)	29	n/a	n/a	n/a	n/a	POS	n/a	2 × 10^8^	n/a	n/a	n/a	n/a	101	329	n/a
Sowithayakasul et al. (30)	30	108	1.6	0.66	57	POS	131	31,132	537	n/a	n/a	788	9	59	130.6
Aquirre-Martinez et al. (31)	31	n/a	n/a	n/a	n/a	POS	n/a	24,320	1,045.1	n/a	n/a	n/a	n/a	n/a	86
Gliwińska et al. (32)	32	101	4.2	1.7	218	n/a	n/a	14.9	363.7	n/a	2.41	n/a	n/a	49.2	414
Sato et al. (33)	33	101	1.6	n/a	1.65	n/a	n/a	927	111.5	n/a	n/a	518	n/a	87	263
	34	50	7.5	n/a	0.23	n/a	n/a	1,401	230.9	n/a	n/a	719	n/a	134	111
	35	101	2.4	n/a	1.23	n/a	n/a	N/A	107.9	n/a	n/a	425	n/a	108	191
	36	96	3.3	n/a	1.21	n/a	n/a	10,115	123.9	n/a	n/a	1,617	n/a	815	118
	37	70	1.0	n/a	1.29	POS	n/a	14,760	282.3	n/a	n/a	1,687	n/a	369	190
	38	113	0.5	n/a	0.98	POS	n/a	2,232	202.6	n/a	n/a	1,110	n/a	194	225
	39	74	3.2	n/a	0.77	POS	n/a	1,127	147.7	n/a	n/a	690	n/a	54	282
	40	108	0.8	n/a	1.59	n/a	n/a	1,780	151.3	n/a	n/a	829	n/a	179	211
	41	91	1.0	n/a	0.71	n/a	n/a	5,213	218.5	n/a	n/a	1,151	n/a	156	212
	42	106	1.0	n/a	0.59	n/a	n/a	1,245	135.4	n/a	n/a	706	n/a	172	55
	43	73	1.7	n/a	0.53	POS	n/a	6,940	169.9	n/a	n/a	3,220	n/a	780	207
Lin et al. (34)	44	90	1.4	0.74	56	n/a	n/a	2,115	n/a	n/a	n/a	n/a	374	995.8	138
Surendran et al. (35)	45	n/a	n/a	n/a	n/a	n/a	n/a	14,969	n/a	n/a	1	1,159	415	1,037	n/a
Cintron et al. (36)	46	90	2.52	n/a	63	POS	n/a	11,329	125	n/a	23	982	n/a	114	190

*Pt, patient; HPC, hemophagocytosis; Hb, hemoglobin; WBC, white blood cells; NEU, neutrophils; PLT, platelets; BM, bone marrow, ESR, erythrocyte sedimentation rate; LDH, leukocyte dehydrogenase, CRP, c reactive protein, AST, aspartate transaminase; ALT alanine aminotransferase; POS positive, NEG negative, n/a not available*.

* *second episode of MAS for the ninth patient; second episode of MAS for the nineteenth patient*.

Considering the debate about the diagnostic criteria of MAS occurring in different rheumatic clinical settings, we analyzed the available clinical experiences on MAS in pSLE by applying the validated EULAR/ACR diagnostic criteria for MAS in patients with sJIA, as schematically represented in Table 3.

**Table 3:** Fulfillment of the sJIA EULAR/ACR 2016 diagnostic criteria for MAS in reported cases of pSLE with MAS.

**Patients**	**Fever**	**Ferritin (>684 ng/mL)**	**Platelet (≤181 × 10^**9**^/L)**	**AST (>48 U/L)**	**Triglycerides (>156 mg/dL)**	**Fibrinogen (≤360 mg/dL)**	**Ref**.	**EULAR/ACR criteria (fulfillment)**
1	+	+	–	+	n/a	n/a	(13)	?
2	+	+	+	n/a	n/a	n/a	(14)	?
3	+	n/a	n/a	n/a	–	n/a	(15)	?
4	+	+	+	+	+	+	(16)	Y
5	+	+	+	+	+	+	(17)	Y
6	+	+	+	–	+	n/a	(18)	Y
7	+	n/a	+	+	+	n/a	(18)	?
8	+	n/a	+	+	+	n/a	(18)	?
9	+	n/a	+	+	+	n/a	(18)	?
9^*^	+	+	+	+	+	n/a	(18)	Y
10	+	n/a	+	–	+	n/a	(18)	?
11	+	+	–	+	+	n/a	(18)	Y
12	+	n/a	+	+	+	n/a	(18)	?
13	+	n/a	+	+	–	n/a	(18)	?
14	+	+	+	+	+	n/a	(18)	Y
15	+	+	+	–	+	n/a	(18)	Y
16	+	+	–	+	+	–	(19)	Y
17	+	n/a	+	–	+	n/a	(20)	?
18	+	n/a	+	+	+	n/a	(20)	?
19	+	+	+	+	+	+	(21)	Y
19^*^	+	+	–	+	+	N/A	(21)	Y
20	+	n/a	+	n/a	n/a	–	(22)	?
21	+	+	+	+	n/a	n/a	(23)	Y
22	+	+	+	+	+	n/a	(24)	Y
23	+	+	+	+	+	n/a	(25)	Y
24	+	+	+	+	+	n/a	(25)	Y
25	+	–	+	–	+	–	(26)	N
26	+	+	+	+	–	–	(26)	Y
27	+	+	+	+	+	n/a	(27)	Y
28	+	+	+	+	+	+	(28)	Y
29	+	+	n/a	+	n/a	n/a	(29)	?
30	+	+	+	+	+	+	(30)	Y
31	+	+	n/a	n/a	+	+	(31)	Y
32	+	–	–	+	+	–	(21)	N
33	+	+	+	+	–	+	(32)	Y
34	+	+	+	+	+	+	(33)	Y
35	+	n/a	+	+	–	+	(33)	?
36	+	+	+	+	–	+	(33)	Y
37	+	+	+	+	+	+	(33)	Y
38	+	+	+	+	+	+	(33)	Y
39	+	+	+	+	–	+	(33)	Y
40	+	+	+	+	–	+	(33)	Y
41	+	+	+	+	+	+	(33)	Y
42	+	+	+	+	–	+	(33)	Y
43	+	+	+	+	+	+	(33)	Y
44	+	+	+	+	n/a	+	(34)	Y
45	+	+	n/a	+	+	+	(35)	Y
46	+	+	+	+	-	+	(36)	Y

*n/a, not available; AST, aspartate transaminase; Ref., reference number; Y, yes; N, no; ?, uncertain (the fulfillment of diagnostic criteria cannot be positively or negatively concluded because of the lack of complete information)*.

* *second episode of MAS for the ninth patient; second episode of MAS for the nineteenth patient*.

In addition to case reports and series, we also analyzed the four [actually five, including the paper by Sato et al. (34)] clinical studies providing aggregated data on clinical and laboratory characteristics of MAS occurring in pSLE patients (11, 33, 37–39). Those articles providing aggregated data for both adult and pediatric patients (without any chance to assess them separately) were excluded. The main laboratory aspects with diagnostic relevance from each study have been extracted and reported in Table 4. Importantly, one of these papers (by Parodi et al.) (37) proposed a set of preliminary criteria to diagnose MAS in patients with a consolidated diagnosis of pSLE: thus, we also challenged the MAS episodes described in the case reports/series with this set of preliminary criteria, as showed in Table 5.

**Table 4:** Relevant laboratory parameters for MAS diagnosis in pSLE patients, as reported in the available clinical studies.

**References laboratory parameters**	**Parodi et al. (37)^**A**^**	**Aytaç et al. (38)^**B**^**	**Borgia et al. (11)^**C**^**	**Sato et al. (33)^**D**^**	**Gerstein et al. (39)^**E**^ [cohort 1]**	**Gerstein et al. (39)^**E**^ [cohort 2]**
Fever (/total patients)	34/38 (89.5%)	6/6 (100%)	38/38 (100%)	11/11 (100%)	10/10 (100 %)	10/10 (100 %)
Ferritin (ng/mL)	2,840.9 ± 3,892.4	4,158 (1,300–15,456)	2,453 (1,072–5,516)	2,006 (927–14,760)	7,579 ± 16,647	2,796 ± 2,164
WBC (10^9^/L)	3.4 ± 2.1	2.45 (0.8–11.3)	2.15 (1.6–2.9)	1.6 (0.5–7.5)	2.6 ± 1.7	2.2 ± 1.3
Hb (g/dL)	7.9 ± 1.6	9.05 (7–11.3)	9.4 (8.4–10.7)	9.6 (5–11.3)	9.7 ± 1.8	9.6 ± 1.8
PLT (10^9^/L)	119.7 ± 91.3	140.5 (63–390)	140 (107–166)	98 (23–165)	158 ± 127	115 ± 54
AST (U/L)	246.5 ± 284.4	53 (22–756)	123 (73–247)	123 (73–247)	163 ± 171	190 ± 204
ALT (U/L)	162.9 ± 233.3	63 (26–107)	83 (50–137)	172 (54–815)	78 ± 69	77 ± 57
LDH (U/L)	1,064.2 ± 1,277.5	836 (321–1,852)	2,186 (1,189–3,092)	829 (425–3,220)	2,094 ± 1,348	2,046 ± 1,019
Triglycerides (mg/dL)	413.5 ± 325.6	235 (65–430)	212.4 (168.15–300.9)	152 (108–283)	194 ± 132	282 ± 108
Fibrinogen (mg/dL)	213 ± 103	313.5 (226–507)	280 (210–340)	199 (55–282)	330 ± 140	270 ± 70
CRP (mg/L)	n/a	8.8 (1.9–15.8)	18.4 (1.9–48.5)	4.6 (0.1–38.7)	21.8 ± 39.7	30.6 ± 34.3
ESR (mm/h)	n/a	29 (4–70)	64 (32–103)	58 (16–143)	81 ± 43	70 ± 52
Sodium (mEq/L)	133.3 ± 6.8	135.5 (132–144)	135 (134–139)	n/a	139 ± 5	136 ± 5

*ESR, erythrocyte sedimentation rate; LDH, leukocyte dehydrogenase, CRP, c-reactive protein, AST, aspartate transaminase; ALT alanine aminotransferase, n number of patients*.

A *Values are reported as mean ± standard deviation*.

B *Values are reported as median value (minimum value-maximum value)*.

C *Values are reported as median value (interquartile range)*.

D *Values are reported as median value (minimum value-maximum value)*.

E *Values are reported as mean value ± standard deviation*.

**Table 5:** Fulfillment of the preliminary criteria by Parodi et al. (37) (for the diagnosis of MAS in pSLE patients) in the reported cases of MAS complicating pSLE.

**Pt**.	**Clinical criteria**					**Laboratory criteria**						**Histology**	**Parodi et al. (37) criteria (fulfilled)**
	**Fever (>38^**°**^C)**	**Hepatomegaly**	**Splenomegaly**	**Hemorrhagic manifestations**	**CNS dysfunction**	**Cytopenia**	**AST (>40 U/L)**	**LDH (>567 U/L)**	**Fibrinogen (≤1.5 g/L)**	**Triglycerides (>178 mg/dL)**	**Ferritin (>500 μg/L)**	**HPH**	
1	+	–	–	n/a	–	–	+	+	n/a	n/a	+	–	Y
2	+	+	+	n/a	n/a	+	n/a	+	n/a	n/a	+	–	Y
3	+	+	–	n/a	n/a	n/a	n/a	–	n/a	–	–	+	?
4	+	–	–	+	+	+	+	+	–	+	+	+	Y
5	+	+	+	n/a	+	+	+	n/a	+	+	+	–	Y
6	+	–	n/a	+	+	+	+	n/a	n/a	+	+	+	Y
7	+	–	n/a	–	+	+	+	n/a	n/a	+	n/a	–	Y
8	+	–	n/a	–	–	+	+	n/a	n/a	+	n/a	–	Y
9	+	+	n/a	–	+	+	+	n/a	n/a	+	n/a	–	Y
9^*^	+	+	n/a	–	+	+	+	n/a	n/a	+	+	+	Y
10	+	+	n/a	–	+	–	–	n/a	n/a	+	n/a	–	?
11	+	–	n/a	–	–	–	+	n/a	n/a	+	+	–	Y
12	+	+	n/a	–	–	+	+	n/a	n/a	+	n/a	–	Y
13	+	–	n/a	–	–	+	+	n/a	n/a	–	n/a	–	Y
14	+	–	n/a	–	+	+	+	n/a	n/a	+	+	–	Y
15	+	–	n/a	+	–	+	–	n/a	n/a	+	+	n/a	Y
16	+	+	n/a	n/a	n/a	+	+	+	–	+	+	–	Y
17	+	+	+	n/a	n/a	+	–	+	n/a	+	n/a	+	Y
18	+	+	+	n/a	n/a	+	+	+	n/a	–	n/a	+	Y
19	+	n/a	n/a	+	+	+	+	+	+	+	+	+	Y
19^*^	+	n/a	n/a	n/a	n/a	–	+	n/a	+	+	+	+	Y
20	+	+	+	+	n/a	+	n/a	n/a	–	n/a	n/a	n/a	?
21	+	n/a	n/a	n/a	n/a	n/a	+	n/a	n/a	n/a	+	n/a	Y
22	+	+	–	n/a	n/a	n/a	+	+	n/a	+	+	–	Y
23	+	+	+	n/a	+	–	+	+	n/a	+	+	+	Y
24	+	+	+	n/a	n/a	+	+	+	n/a	+	+	–	Y
25	+	+	+	+	+	+	n/a	+	–	+	+	+	Y
26	+	+	+	n/a	+	+	+	+	–	n/a	+	+	Y
27	–	–	–	n/a	n/a	+	+	+	n/a	+	+	+	N
28	–	n/a	n/a	n/a	n/a	+	+	n/a	+	+	+	+	?
29	+	+	–	+	–	n/a	+	n/a	n/a	n/a	+	+	Y
30	+	–	+	n/a	n/a	+	+	+	+	+	+	+	Y
31	+	+	+	n/a	n/a	n/a	n/a	n/a	n/a	+	+	+	Y
32	+	n/a	+	n/a	+	–	+	n/a	–	+	–	n/a	Y
33	+	–	–	n/a	n/a	+	+	–	–	–	+	n/a	Y
34	+	–	–	n/a	n/a	+	+	+	+	+	+	n/a	Y
35	+	+	+	n/a	n/a	+	+	–	–	–	n/a	n/a	Y
36	+	–	–	n/a	n/a	+	+	+	+	–	+	n/a	Y
37	+	–	+	n/a	n/a	+	+	+	–	+	+	+	Y
38	+	–	+	n/a	n/a	+	+	+	–	+	+	+	Y
39	+	+	+	n/a	n/a	+	+	+	–	–	+	+	Y
40	+	–	–	n/a	n/a	+	+	+	–	–	+	n/a	Y
41	+	–	–	n/a	n/a	+	+	+	–	+	+	n/a	Y
42	+	–	–	n/a	n/a	+	+	+	+	–	+	n/a	Y
43	+	–	–	n/a	n/a	+	+	+	–	–	+	+	Y
44	+	n/a	n/a	n/a	n/a	+	+	n/a	+	n/a	+	n/a	Y
45	+	n/a	n/a	n/a	+	+	+	+	+	+	+	n/a	Y
46	+	n/a	n/a	+	+	+	+	+	-	-	+	+	Y

*HPH, hemophagocytosis; n/a, not available; Y, yes; N, no; ?, uncertain (the fulfillment of this set of diagnostic criteria cannot be positively or negatively concluded, due to the lack of complete information); CNS, central nervous system; LDH, leukocyte dehydrogenase; AST, aspartate transaminase*.

* *second episode of MAS for the ninth patient*;

* *second episode of MAS for the nineteenth patient*.

## Discussion

MAS is a severe and acute complication of several systemic inflammatory diseases: indeed, it can result in a progressive multi-organ failure and, if not timely recognized and treated, it may lead to a fatal outcome (40). As previously mentioned in the introduction, the occurrence of MAS may be underestimated in pSLE and several other rheumatic diseases: the absence of validated sets of disease-specific criteria to diagnose MAS definitely contributes to this situation (11).

No matter the particular clinical background, the diagnosis of MAS was originally based on the same diagnostic criteria used for primary HLH (HLH-2004 classification system), which include clinical, laboratory and histopathologic findings (fever, splenomegaly, cytopenia, elevated triglycerides/decreased fibrinogen, decreased NK cell function, increased ferritin, and increased soluble IL-2 receptor levels, demonstration of hemophagocytosis) (41). More recently, the EULAR/ACR provided a new set of diagnostic criteria for MAS validated in sJIA patients: this complication is diagnosed whenever a febrile patient with known or suspected sJIA shows high serum ferritin (>684 ng/ml) plus any two of the following criteria: platelet count <181^*^109/l, aspartate aminotransferase >48 U/l, triglycerides >156 mg/dl, fibrinogen <360 mg/dl (12).

In this review, we tried to analyze the potential applicability and performance of these EULAR/ACR criteria for sJIA-related MAS in patients affected with pSLE. Therefore, we extracted the main (clinical and laboratory) individual data provided by all the available case reports and series of children developing MAS in the context of SLE, as showed in Tables 1 and 2.

Through this literature research approach, we collected a pool of 46 pediatric patients affected with pSLE-related MAS (13–36). Most of them were female (*n* = 38, 82.6%) and their mean age was around 12 years (age range: 4–17 years): both these demographic aspects reflect the gender and age distribution of pSLE. Importantly, only 9 out of 35 patients (25.7%) are clearly reported as having a diagnosis of pSLE at the time (and, thus, in consequence) of MAS occurrence: in these cases, this complication appeared after a variable amount of time, ranging from <1 months to more than 4 years. All the remaining 26 patients received the diagnosis of pSLE after MAS occurrence. The 11 patients described by Sato et al. (33) may have developed MAS at the pSLE onset or in the early disease phases; however, the authors do no provide precise information on this specific aspect. The therapeutic approach was variable, but this discussion is beyond the purpose of this article; however, 6 pSLE patients (13.0%) died because of MAS and, interestingly, 4 of them had been already diagnosed with pSLE, which further supports the need of reliable diagnostic criteria in this rheumatic disease.

As showed in Table 3, we tried to apply the aforementioned JIA-related EULAR/ACR MAS criteria to each of these 48 episodes of pSLE-related MAS. Unfortunately, not all case presentations provided the full set of laboratory data included in this diagnostic classification; however, a sufficient information to conclude whether the EULAR/ACR criteria are fulfilled or not, was available for most cases (*n* = 33, 68.7%). Among them, only 2 cases out of 33 (6.1%) did not fulfill these criteria for sure, because of a ferritin value lower than the 684 mg/ml cut-off (pt. 25: 592 ng/ml; pt. 32: 540 ng/ml); otherwise, both cases would have satisfied the additional laboratory criteria. As regards the other 15 episodes (31.3%) with insufficient laboratory data, the ferritin value is missing in 12 cases, which precludes by itself the assessment and confirmation of MAS diagnosis, according to the EULAR/ACR criteria. However, it is worth to note that, whenever the other laboratory parameters were reported, those would have been consistent with MAS diagnosis, if the ferritin value had been available and greater than the cut-off. In the remaining 3 cases, whose ferritin values were reported (and it was >684 mg/ml), at least three other laboratory parameters of the EULAR/ACR criteria were unknown, impairing any further diagnostic conclusions in this regard.

The ACR/EULAR criteria validated to diagnose sJIA-related MAS seem to be applicable to a clinical picture of persistent fever associated or leading to a diagnosis of pSLE, overall. Some concerns may be related to the ferritin cut-off: indeed, among the only two episodes which did not satisfy this criterion, the ferritin value was elevated anyway and, thus, was able to suggest the diagnosis of MAS, especially in light of the fulfillment of the other laboratory parameters included in ACR/EULAR diagnostic system. Of course, some laboratory analysis-related factors and/or the timing of the samples collection may have affected this result in these two patients. However, for one patient (pt. 32), the article reported two different (2–3 weeks apart) measurements of ferritin and, in both circumstances, the ferritin value was <684 mg/ml, even though was higher than the upper limit of the normal range (540 and 596 ng/ml; normal range: 20–200 ng/ml).

This systematic review also included those few clinical studies (n = 5) providing aggregated data specific for pediatric patients with pSLE and MAS (11, 33, 37–39).

In 2009, Parodi et al. described 38 patients with ascertained diagnosis of pSLE, who eventually developed MAS (37). Here, the amplitude of the standard deviation of the laboratory parameters suggested a great variability that may not fulfill the ACR/EULAR criteria. In detail, the pooled ferritin value resulted to be 2,840.9 ± 3,892.4 ng/ml, which suggests that the 684 mg/ml cut-off is likely not to be reached in all these patients. Importantly, in this paper, the authors proposed some “preliminary” diagnostic criteria for MAS as a complication of pSLE: therefore, we also re-analyzed the aforementioned 48 pSLE-related MAS episodes (extracted from case reports and series) accordingly, as showed in Table 5 (where these preliminary criteria are listed). Interestingly, the lower ferritin cut-off (>500 ng/ml) in this set of diagnostic criteria would have allowed to make a MAS diagnosis even in those aforementioned 2 patients (out of 33) who did not fulfill the ACR/EULAR classification system.

This point seems to be supported by the selected clinical studies, as discussed below. In 2012, Bennet et al. compared the clinical characteristics of MAS between 19 patients with pSLE and 102 patients with JIA (42). Unfortunately, these authors did not provide any clinical and laboratory data: therefore, this study was not included in our literature research output. Conversely, this information was provided in a similar study by Aytaç et al. (38). These authors compared 31 sJIA-related MAS and 6 pSLE-related MAS episodes: it is important to notice that the ferritin values were remarkably higher in sJIA patients than in pSLE patients [7,838 (360–150,099) ng/ml vs. 4,158 (1,300–15,456) ng/ml, respectively], which may support the fact that lower ferritin values can be expected in patients with pSLE-related MAS, due to a different inflammatory background compared to sJIA, probably.

Borgia et al. described 38 pSLE patients with MAS (representing 9% of a cohort of 403 children affected with SLE). First, 68% of these patients had a concomitant diagnosis of MAS and pSLE, indicating that a previous diagnosis of pSLE was present in 32% of cases only, which is consistent with our analysis of case reports/series. Here, the median value of ferritin was 2,453 (IQR: 1,072–5,516) ng/ml; however, the authors specified that they reported the most abnormal laboratory value before MAS treatment (11). In the study by Sato et al. among 46 “new-onset jSLE patients,” 11 patients with MAS (whose data were provided in individual form as well) and 19 patients without MAS were compared. As mentioned, all 10 MAS patients (whose ferritin level was available) had values definitely higher than 684 mg/ml [2,006 (927–14,760) ng/ml]. (33). Finally, Gerstein et al. recently described two different cohorts of SLE children diagnosed in their center, based on the period of pSLE diagnosis (2003–2007: *n* = 34; 2008–2013: *n* = 41). Overall, they observed 20 episodes of MAS (10 episodes in each cohort): the ferritin values resulted 7,579 ± 16,647 and 2,796 ± 2,164 ng/ml, respectively. Based on this experience, they suggested a ferritin cut-off of 669 ng/ml as reliable to diagnose MAS in their two cohorts (39).

Therefore, when considering a diagnosis of MAS in children without any clear rheumatic disorder yet (and, thus, potentially affected with pSLE), it may be useful to consider this set of diagnostic criteria by Parodi et al. (37) (although those have not received a complete validation yet), rather than applying the HLH-2004 or the sJIA-related ACR/EULAR criteria.

Finally, it is important to mention the limitations of our systematic review. First, the case presentations were quite heterogeneous, as regards the description of the clinical aspects and laboratory parameters, which has not allowed to complete our analysis for all MAS episodes. Moreover, the timing of the laboratory assessment was not clearly reported for each clinical case and it is likely that there was a remarkable variability in this aspect, which may have affected the value of some diagnostic parameters, including serum ferritin. However, despite some limitations, our analysis and discussion confirmed and emphasized the importance of having specific diagnostic criteria for MAS according to the different clinical and rheumatological settings.

## Conclusion

In conclusion, MAS is not a very rare complication of pSLE and it is characterized by several diagnostic challenges, which could lead to delayed diagnosis and/or under-estimation of this complication. Although persistent and/or septic-like fever variably associated to other clinical manifestations (e.g., hepatosplenomegaly, lymphadenopathy, etc.) could suggest the diagnosis of MAS, the fact that this condition can occur before a diagnosis of pSLE has already made, may significantly hamper the timely recognition of MAS.

Indeed, MAS often occurs at the onset of pSLE. Importantly, specific criteria should be considered for MAS diagnosis according to different rheumatic settings. As regards pSLE, the preliminary criteria by Parodi et al. (37) (which were set in patients with an existing diagnosis of pSLE) seem to perform better than the sJIA-related ACR/EULAR criteria (because of a lower ferritin cut-off), even in patients who receives a diagnosis of pSLE after developing an episode of MAS as an initial manifestation of this rheumatic disease.

## Data Availability Statement

The original contributions presented in the study are included in the article/supplementary material, further inquiries can be directed to the corresponding author/s.

## Author Contributions

DP conceived the study. AA performed the systematic literature research and carried out the identification and screening of the literature records. AA, VS, and DP assessed the eligible articles. AA drafted the figures and tables. AA, VS, and DP contributed to the manuscript draft. DP wrote the final manuscript and contributed to refine figures and tables. DP, DA, ZM, and MA provided substantial intellectual contribution. All authors contributed to the article and approved the submitted version.

## Conflict of Interest

The authors declare that the research was conducted in the absence of any commercial or financial relationships that could be construed as a potential conflict of interest.

## References

[1] Kiriakidou M Ching CL. systemic lupus erythematosus. Ann Intern Med. (2020) 172:ITC81–96. 10.7326/AITC202006020 32479157

[2] Pons-Estel GJ Alarcón GS Scofield L Reinlib L Cooper GS. Understanding the epidemiology and progression of systemic lupus erythematosus. Semin. Arthritis Rheum. (2010) 39:257–68. 10.1016/j.semarthrit.2008.10.007 19136143 PMC2813992

[3] Danchenko N Satia JA Anthony MS. Epidemiology of systemic lupus erythematosus: a comparison of worldwide disease burden. Lupus. (2006) 15:308–18. 10.1191/0961203306lu2305xx 16761508

[4] Kamphuis S Silverman ED. Prevalence and burden of pediatric-onset systemic lupus erythematosus. Nat Rev Rheumatol. (2010) 6:538–46. 10.1038/nrrheum.2010.121 20683438

[5] Sahin S Adrovic A Barut K Canpolat N Ozluk Y Kilicaslan I. Juvenile systemic lupus erythematosus in Turkey: demographic, clinical and laboratory features with disease activity and outcome. Lupus. (2018) 27:514–9. 10.1177/0961203317747717 29233038

[6] Bundhun PK Kumari A Huang F. Differences in clinical features observed between childhood-onset versus adult-onset systemic lupus erythematosus: a systematic review and meta-analysis. Medicine. (2017) 96:80–6. 10.1097/MD.0000000000008086 28906413 PMC5604682

[7] Sahin S Adrovic A Barut K Durmus S Gelisgen R Uzun H. Pentraxin-3 levels are associated with vasculitis and disease activity in childhood-onset systemic lupus erythematosus. Lupus. (2017) 26:1089–94. 10.1177/0961203317699286 28420068

[8] Gavand PE Serio I Arnaud L Costedoat-Chalumeau N Carvelli J Dossier A. Clinical spectrum and therapeutic management of systemic lupus erythematosus-associated macrophage activation syndrome: a study of 103 episodes in 89 adult patients. Autoimmun Rev. (2017) 16:743–9. 10.1016/j.autrev.2017.05.010 28483541

[9] Poddighe D Dauyey K. Macrophage activation syndrome in juvenile dermatomyositis: a systematic review. Rheumatol Int. (2020) 40:695–702. 10.1007/s00296-019-04442-1 31529231

[10] Grom AA Mellins ED. Macrophage activation syndrome: advances towards understanding pathogenesis. Curr Opin Rheumatol. (2010) 22:561–6. 10.1097/01.bor.0000381996.69261.71 20517154 PMC4443835

[11] Borgia RE Gerstein M Levy DM Silverman ED Hiraki LT. Features, treatment, and outcomes of macrophage activation syndrome in childhood-onset systemic lupus erythematosus. Arthritis Rheumatol. (2018) 70:616–24. 10.1002/art.40417 29342508

[12] Ravelli A Minoia F Davì S Horne A Bovis F Pistorio A. 2016 classification criteria for macrophage activation syndrome complicating systemic juvenile idiopathic arthritis: a European league against rheumatism/American college of rheumatology/paediatric rheumatology international trials organisation collaborative initiative. Ann Rheum Dis. (2016) 75:481–9. 10.1136/annrheumdis-2015-208982 26865703

[13] Avčin T Shirley ML Schneider R Ngan B Silverman ED. Macrophage activation syndrome as the presenting manifestation of rheumatic diseases in childhood. J Pediatr. (2006) 148:683–6. 10.1016/j.jpeds.2005.12.070 16737887

[14] McCann LJ Hasson N Pilkington CA. Macrophage activation syndrome as an early presentation of lupus. J Rheumatol. (2006) 33:438–40. 16482659

[15] Rajam L Prasad V Yatheesha BL. Reactive hemophagocytic syndrome. Indian J Pediatr. (2008) 75:1261–3. 10.1007/s12098-008-0170-y 18810357

[16] Yeap ST Sheen JM Kuo HC Hwang KP Yang KD Yu HR. Macrophage activation syndrome as initial presentation of systemic lupus erythematosus. Pediatr Neonatol. (2008) 49:39–42. 10.1016/S1875-9572(08)60010-8 18947015

[17] Zulian F Piccinini P Martini G Jorini M de Benedictis FM. Macrophage activation syndrome as trigger event for systemic lupus erythematosus in children. J Paediatr Child Health. (2009) 45:621–2. 10.1111/j.1440-1754.2009.01582 19825028

[18] Campos LM Omori CH Lotito AP Jesus AA Porta G Silva CA. Acute pancreatitis in juvenile systemic lupus erythematosus: a manifestation of macrophage activation syndrome? Lupus. (2010) 19:1654–8. 10.1177/0961203310378863 20837568

[19] Gharib B Ziaee V Moradinejad MH Esmaeili S. Pleuritic chest pain; where should we search for? Iran J Pediatr. (2011) 21:557–62. 23056851 PMC3446146

[20] Lin CI Yu HH Lee JH Wang LC Lin YT Yang YH. Clinical analysis of macrophage activation syndrome in pediatric patients with autoimmune diseases. Clin Rheumatol. (2012) 31:1223–30. 10.1007/s10067-012-1998-0 22615046

[21] Vilaiyuk S Sirachainan N Wanitkun S Pirojsakul K Vaewpanich J. Recurrent macrophage activation syndrome as the primary manifestation in systemic lupus erythematosus and the benefit of serial ferritin measurements: a case-based review. Clin Rheumatol. (2013) 32:899–904. 10.1007/s10067-013-2227-1 23483294

[22] Jiménez AT Vallejo ES Cruz MZ Cruz AC Jara BS. Macrophage activation syndrome as the initial manifestation of severe juvenile onset systemic lupus erythematosus. Favorable response to cyclophosphamide. Reumatol Clin. (2014) 10:331–5. 10.1016/j.reumae.2013.12.009 24035795

[23] Nakagishi Y Shimizu M Kasai K Miyoshi M Yachie A. Successful therapy of macrophage activation syndrome with dexamethasone palmitate. Mod Rheumatol. (2016) 26:617–20. 10.3109/14397595.2014.906053 24754272

[24] Noh JH Jeong DY Jeon IS Kim HM. Macrophage activation syndrome triggered by herpes viral infection as the presenting manifestation of juvenile systemic lupus erythematosus. Pediatr Infect Vaccine. (2015) 22:210–5. 10.14776/piv.2015.22.3.210

[25] Gupta D Mohanty S Thakral D Bagga A Wig N Mitra DK. Unusual association of hemophagocytic lymphohistiocytosis in systemic lupus erythematosus: cases reported at tertiary care center. Am J Case Rep. (2016) 17:739–44. 10.12659/AJCR.899433 27733745 PMC5065291

[26] Alkoht A Hanafi I Khalil B. Macrophage activation syndrome: a report of two cases and a literature review. Case Rep Rheumatol. (2017) 2017:5304180 10.1155/2017/5304180 29209549 PMC5676417

[27] Casciato A Lind C Olson APJ Binstadt BA Davis AM. A howling cause of pancytopenia. J Hosp Med. (2018) 13:205–9. 10.12788/jhm.2855 29069118

[28] Guru S Behera A Debta N Kumar R. Systemic lupus erythematosus presenting as macrophage activation syndrome. J Clin Diagn Res. (2018) 12:OD06–7. 10.7860/JCDR/2018/31033.1126130480003

[29] Moideen S Uvais NA. Systemic lupus erythematosus presenting as immunoglobulin responsive macrophage activation syndrome. Arch Rheumatol. (2019) 34:117–8. 10.5606/ArchRheumatol.2019.6936

[30] Sowithayasakul P Pruangprasert P Takkinsatian P Sinlapamongkolkul P. Macrophage activation syndrome presenting in a child with concomitant systemic lupus erythematosus and HIV infection: a case report. Thammasat Med J. (2018) 18:434–9.

[31] Aguirre-Martinez I Vélez-Tirado N García-Romero MT Rodríguez-Lozano AL Corcuera-Delgado CT Yamazaki-Nakashimada M. Rowell syndrome complicated with macrophage activation syndrome in a child. Lupus. (2019) 28:1716–21. 10.1177/0961203319886030 31674268

[32] Gliwińska A Bjanid O Adamczyk P Czubilińska-Łada J Dzienniak A Morawiecka-Pietrzak M. A rare complication of systemic lupus erythematosus in a 9-year-old girl: answers. Pediatr Nephrol. (2020) 35:781–5. 10.1007/s00467-019-04411-7 31823043 PMC7096361

[33] Sato S Uejima Y Arakawa Y Furuichi M Suganuma E Fujinaga S. Clinical features of macrophage activation syndrome as the onset manifestation of juvenile systemic lupus erythematosus. Rheumatol Adv Pract. (2019) 3:1–6. 10.1093/rap/rkz013 31432001 PMC6649928

[34] Lin Q Zhang M Tang H Shen Y Zhu Y Xu Q. Acute pancreatitis and macrophage activation syndrome in pediatric systemic lupus erythematosus: case-based review. Rheumatol Int. (2020) 40:811–9. 10.1007/s00296-019-04388-4 31377830

[35] Surendran S George T Balan S Easwar SV. Staring at an abscess, but lupus stares back. J R Coll Phys Edinb. (2020) 50:299–302. 10.4997/JRCPE.2020.318 32936108

[36] Cintron D Rodríguez-Agramonte JC Soto-Velez LA. Altered mental status in a 16-year-old adolescent male. Cureus. (2020) 12:e11664. 10.7759/cureus.11664 33391902 PMC7769498

[37] Parodi A Davì S Pringe AB Pistorio A Ruperto N Magni-Manzoni S. Macrophage activation syndrome in juvenile systemic lupus erythematosus: a multinational multicenter study of thirty-eight patients. Arthritis Rheum. (2009) 60:3388–99. 10.1002/art.24883 19877067

[38] Aytaç S Batu ED Ünal S Bilginer Y Çetin M Tuncer M. Macrophage activation syndrome in children with systemic juvenile idiopathic arthritis and systemic lupus erythematosus. Rheumatol Int. (2016) 36:1421–9. 10.1007/s00296-016-3545-9 27510530

[39] Gerstein M Borgia RE Dominguez D Feldman BM Liao F Levy D. Predicting macrophage activation syndrome (MAS) in childhood-onsetsystemic lupus erythematosus (cSLE) patients at diagnosis. J Rheumatol. (2020). 10.3899/jrheum.200292. [Epub ahead of print]. 33262295

[40] Ravelli A Davì S Minoia F Martini A Cron RQ. Macrophage activation syndrome. Hematol Oncol Clin North Am. (2015) 29:927–41. 10.1016/j.hoc.2015.06.010 26461152

[41] Henter JI Horne A Aricó M Egeler RM Filipovich AH Imashuku S. HLH-2004: diagnostic and therapeutic guidelines for hemophagocytic lymphohistiocytosis. Pediatr Blood Cancer. (2007) 48:124–31. 10.1002/pbc.21039 16937360

[42] Bennett TD Fluchel M Hersh AO Hayward KN Hersh AL Brogan TV. Macrophage activation syndrome in children with systemic lupus erythematosus and children with juvenile idiopathic arthritis. Arthritis Rheum. (2012) 64:4135–42. 10.1002/art.34661 22886474 PMC3505557

